# The Effects of Daily Consumption of Functionalized Yogurts with Sacha Inchi Oil and Interspecific Hybrid Palm Oil on the Lipid Profile and ApoB/ApoA1 Ratio of Healthy Adult Subjects

**DOI:** 10.3390/foods13233973

**Published:** 2024-12-09

**Authors:** Ruby-Alejandra Villamil, Laura-Natalia Romero, Juan-Pablo Ruiz, Diana-Cristina Patiño, Luis-Felipe Gutiérrez, Lilia-Yadira Cortés

**Affiliations:** 1 Departamento de Nutrición y Bioquímica, Pontificia Universidad Javeriana, Bogotá 110231, Colombia; ln.romero@javeriana.edu.co (L.-N.R.); ruizcjuan@javeriana.edu.co (J.-P.R.); ycortes@javeriana.edu.co (L.-Y.C.); 2 Departamento de Microbiología, Pontificia Universidad Javeriana, Bogotá 110231, Colombia; dpatino@javeriana.edu.co; 3 Instituto de Ciencia y Tecnología de Alimentos, Universidad Nacional de Colombia, Bogotá 111311, Colombia; lfgutierreza@unal.edu.co

**Keywords:** yogurt, PUFA, cardiovascular risk, lipid profile, healthy volunteers

## Abstract

Sacha Inchi oil (SIO) and hybrid palm oil (HPO) are potential sources of unsaturated fatty acids to improve the lipid profile of dairy products. This study evaluated, for the first time, the effects of the daily consumption of yogurts with enhanced fatty acid profiles on plasma lipids related to cardiovascular disease (CVD) risk factors. A pilot, randomized, double-blind, parallel-controlled trial was conducted with 47 participants assigned to three groups: SIO-enriched yogurt (Group A), HPO-enriched yogurt (Group B), and plain yogurt (Group C). Fasting blood samples were collected at baseline and after 1, 2, and 3 months to measure plasma lipids (TC, LDL-C, HDL-C, and TAG), ApoA1, and ApoB. While no significant changes were observed in the overall lipid profiles, notable within-group effects were identified. The total cholesterol (TC) dropped by 2.8%, 1.3%, and 3.3%, and LDL-C by 1.6%, 2.5%, and 2% in Groups A, B, and C, respectively. Additionally, the intake of monounsaturated fatty acids (MUFA), polyunsaturated fatty acids (PUFA), and vitamin E significantly increased in Groups A and B. These results suggest that SIO and HPO can be used as milk fat substitutes to enhance the nutritional profile of yogurts without affecting CVD biomarkers in healthy individuals.

## 1. Introduction

Cardiovascular disease (CVD) is the leading cause of morbidity and mortality worldwide; in the Americas, CVD has the highest death toll compared to the other world regions [1]. In Colombia, out of 100,000 people, 130.4 deaths were due to CVD in 2019 [1]. Furthermore, the recent health emergency caused by COVID-19 has worsened the clinical outcomes in people with pre-existing CVD [2].

The development of CVD is determined by non-modifiable and modifiable factors; diet is one of the latter and its management seeks to reduce CVD [3]. This poses an opportunity for the food industry to play a more active role in CVD risk reduction by designing and processing functional food (FF) items with enhanced shares of CVD-protective food components.

The so-called Western diet is a major risk factor for CVD due to its high share of processed foods, which entails considerable quantities of saturated fatty acid (SFA), *trans* fatty acids, sugar, and sodium and lacks nutrients and bioactive phytochemicals that promote cardiovascular health, such as unsaturated fatty acids (UFA), vitamin E, and antioxidants [3]. The main food sources of unsaturated fatty acids are oils from vegetable and animal origin (olives, canola, flaxseed, fish, algae, among others), which are already available for mass consumption [4]. In Colombia, two locally available oils rich in unsaturated fatty acids are those obtained from Sacha Inchi (*Plukenetia volubilis* L.) (Sacha Inchi oil—SIO) and from hybrid palm (*Elaeis oleifera × Elaeis guineensis*) cultivars (interspecific hybrid palm oil—HPO). These oils are sources of alpha-linolenic acid (ALA), oleic acid (OA), and vitamin E, respectively [5,6]. 

SIO is nutritionally attractive due to its high (45%) ALA content [7]. ALA is a precursor of eicosapentaenoic acid (EPA) and docosahexaenoic acid (DHA) and is a local, sustainable, and plant-based alternative to marine omega 3 polyunsaturated fatty acids (PUFAs) [8]. Recently, it has been suggested that ALA intake leads to a lower risk of total atherosclerotic CVD in subjects with a low intake of EPA and DHA [8]. Furthermore, clinical trials have shown that ALA has a hypo-lipidemic effect, reduces visceral fat, and ameliorates the inflammatory reaction caused by obesity [9]. HPO also possesses remarkable nutritional qualities due to its content of OA (48–60%), vitamin E (γ-tocotrienol; 40–60% of total tocols), β-carotene (52–60% of total carotenes), and α-carotene (33–36% of total carotenes) content. Recently, HPO has been recognized as the “tropical equivalent of olive oil”, due to its high oleic acid (OA) content [5], which is a monounsaturated fatty acid (MUFA). Replacing fats and oils high in saturated or *trans* fatty acids with oils rich in OA has favorable and comparable effects on plasma lipid risk factors and overall coronary heart disease risk with olive oil [10]. Lastly, tocotrienol is an isoform of vitamin E with higher biological effectiveness than tocopherols in reducing low density lipoprotein cholesterol (LDL-C) by up to 25% [11]. 

Yogurt has been widely studied as an FF due to the diverse properties of the secondary metabolites derived from the lactic acid fermentation by the starter culture [12]. Consequently, yogurt is regarded as a vehicle for bioactive compounds that enhance the health of its consumers. Hence, Babio et al. [13] indicated that yogurt is a diet quality indicator. In contrast to the extensive literature on the enhancement of yogurt’s fatty acid profiles, the effects of the intake of these yogurts on cardiovascular health have been scarcely investigated; however, those trials have demonstrated yogurt’s effectivity in reducing CVD risk via different biological mechanisms [14].

The CVD risk markers commonly assessed are those pertaining to the lipid profile, namely plasma triacylglycerol (TAG), total cholesterol (TC), high-density lipoprotein cholesterol (HDL-C), and LDL-c cholesterol (LDL-c). Furthermore, the ratio between apolipoproteins A1 and B (ApoB/ApoA1 ratio) has emerged as an early CDV risk marker since it is a strong predictor of atherosclerotic cardiovascular disease [15]. The aim of the present study was to examine, for the first time, the effect of the intake of yogurts supplemented with two different UFAs sources (SIO and HPO) on plasma lipids and ApoB/ApoA1 ratios in healthy (CVD-free) individuals. This assessment was performed with a randomized double-blind trial in healthy adult subjects, in which we contrasted the effects on selected CVD-markers of the daily intake of SIO and HPO-supplemented yogurts with those of full-fat yogurt consumption in the total intervention for a three-month period.

## 2. Materials and Methods

### 2.1. Trial Design 

The study was designed as a randomized, double-blind, and parallel-controlled pragmatic trial. The research protocol was approved by the Research and Ethics Committee of the Science Faculty at Pontificia Universidad Javeriana (27 April 2021). Written informed consent was obtained from all participants. 

### 2.2. Participants 

**Figure 1:**
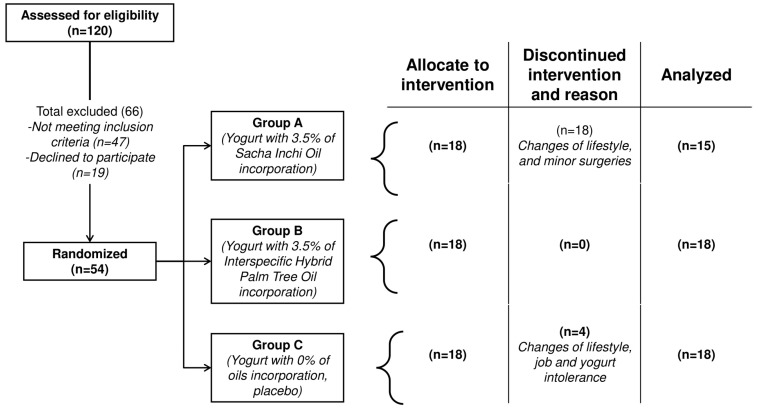
Trial enrollment diagram.

Sample size was determined following a convenience criterion and subjects were stratified by sex and randomized with an unrestricted randomization method to be assigned into one of three groups. Group size was determined considering the literature in which the same CVD markers were evaluated [14]. The Food and Drugs Administration Department postulate was considered, which states that the total number of subjects included in phase 1 studies varies with the drug but is generally in the range of 20 to 80 subjects [16]. Fifty-four subjects were enrolled, and forty-seven of them participated in the study (Figure 1). 

The study was conducted between March and November 2022 (274 days). Subjects were recruited among the staff and students at Pontificia Universidad Javeriana, Bogota-Colombia. The study’s enrollment process ensured a balanced selection of participants between genders, with each group initially comprising 9 males and 9 females. The first inclusion criterium was age; only men aged ≤ 50 years and women aged ≤ 45 years were considered. Moreover, enrolled subjects were healthy, which was assessed by a clinical status survey, with normal total cholesterol (≤200 mg/dL) levels, have a yogurt consumption habit of at least three times a week, a maximum level of moderate physical activity, a body mass index (BMI) of ≤29.9 kg/m^2^), and TAG levels below 400 mg/dL. Exclusion criteria included the following: the presence of chronic disease (HTA, Diabetes Mellitus, among others), a history of illicit drug use and/or chronic alcohol use, active smoking, a well-established allergy to red palm olein and/or milk and its derivatives, consumption of nutritional supplements (antioxidants, omega 3, fiber, phytosterols), pregnancy or lactation, having menopause, and high consumption of nuts, flaxseed, chia, and sesame seeds. 

One hundred and twenty (120) subjects were screened for enrollment in this study, of which fifty four met all inclusion criteria. A total of 47 participants (47% females and 53% males) finished the 12-week intervention period (Group A: 15 subjects, Group B: 18 subjects, and Group C: 14 subjects). Seven participants withdrew from the study because of changes in individual lifestyle habits, yogurt intolerance, and minor surgeries (Figure 1). 

### 2.3. Interventions 

Yogurts were prepared according with our previous study [5] at the Institute of Food Science and Technology at Universidad Nacional de Colombia, Bogota. All eligible subjects were randomly assigned into 3 groups (A, B, and C). Subjects in the three groups were provided with 200 g of 3.5% fat yogurts per day for 3 months (12-weeks, 90 days). Group A subjects were given yogurt with fat from locally produced Sacha Inchi oil; Group B subjects were provided with yogurt containing fat from Colombian hybrid palm oil; and the control (C) group was given yogurt with unreplaced milk fat (standard yogurt). Nutrition facts in a serving size of 200 g of yogurt are shown in Table 1 and its safety information is given in Appendix A and Appendix B. All participants were asked to keep their usual diets, avoiding the intake of other fermented dairy products and maintaining their usual exercise routines. 

### 2.4. Measurement of Anthropometric Parameters and Blood Pressure (BP) 

Anthropometric indices and blood pressure were evaluated at the beginning and end of each intervention. Weight, height, body composition, and BMI were measured and calculated using a 264 scale and mBCA 514 bioimpedance spectroscopy (Seca, Hamburg, Germany). Waist circumference (WC) was measured with a tape measure (Lufkin, Missouri City, TX, USA), while the patients were at the end of breathing out, at the midpoint of lower rib and iliac crest. All the measurements were taken by the same person to decrease intra-observer error. A standardized mercury sphygmomanometer 160 was used to record resting BP in a quiet room, using a digital BP monitor. 

### 2.5. Assessment of Dietary Intake and Physical Activity 

Dietary intake was assessed at the beginning, during, and at the end of the study by a 3-day dietary record and a 24 h recall questionnaire. Food scales and models were also used to enhance portion size. Dietary intakes were analyzed by MenusPlus 8 software (version 2018; Comunidad Web LTDA). Physical activity was also assessed at the beginning and end of the study using the International Physical Activity Questionary-Short Form (IPAQ-SF), which has been recommended as a cost-effective method to assess physical activity. 

### 2.6. Measurement of CVD Markers 

A blood sample was taken at baseline and at months 1, 2, and 3 for each intervention. The plasma lipid profile and apolipoproteins A1 and B (Apo A1 and Apo B) were measured after at least eight hours of fasting. Plasma TC, HDL-c, TAG, and LDL-c levels were determined using an enzymatic colorimetric assay in a spectrophotometer (Beckman Coulter, Sydney, Australia) at 540/600 nm, 600/700 nm, and 660/800 nm. ApoA1 and ApoB were determined by the immunoturbidimetric method, and the ApoB/ApoA1 ratio was estimated. The normal upper limits and ranges for the markers assessed were as follows: for TAG, 1.70 mmol/L; for TC, 5.18 mmol/L; for LDL, 4.12 mmol/L; for HDL, 1.04–1.55 mmol/L; for ApoA1, 1.25–2.15 g/L for women and 1.10–2.05 mmol/L for men; for Apo B, 0.55–1.25 g/L for women and 0.55–1.4 g/L for men; and for ApoB/ApoA1, 0.30–0.9 for women and 0.35–1.00 for men. Samples were collected and processed at the Hospital Universitario San Ignacio (Bogota, Colombia). 

### 2.7. Statistical Analyses 

The Kolmogorov–Smirnov and Shapiro–Wilk tests were applied to ascertain normal distribution of the data. Paired *t*-tests and Wilcoxon signed-rank test were conducted to evaluate marker value differences between baseline and the end of intervention period. To compare data among the three intervention groups, two-way analyses of variance (ANOVA) and Kruskal–Wallis test were performed. Statistical assessment of the data was performed using STATGRAPHICS Centurion XIX software. The biochemical data were analyzed using the Nonparametric Analysis of Longitudinal Data in Factorial Experiments NparLD package in R-project. Data are expressed as mean ± standard deviation (SD) in the text. Intake variables were estimated using MenusPlus 8 (Comunidad Web LTDA, 2022). The statistical significance level was set at 5%. 

## 3. Results

### 3.1. Population Characteristics: Baseline and End Status after the Intervention 

The main characteristics (number of subjects, age, body weight, body mass index, fat mass index, fat-free mass index, waist circumference, and blood pressure) of the study population are outlined in Table 2. The intake of fortified yogurt during three months did not significantly affect (*p* < 0.05) the participants’ body composition parameters and blood pressure when compared with their baseline values. This was true in men and women alike. However, in the placebo (Group C), a significant difference (*p* ≤ 0.05) between baseline (122.9 ± 8.3) and the study end (115.4 ± 10.6) was detected for blood systolic pressure, which was slightly higher in males than in females. 

### 3.2. Cardiovascular Disease Risk Markers

#### 3.2.1. Lipid Profile 

**Figure 2:**
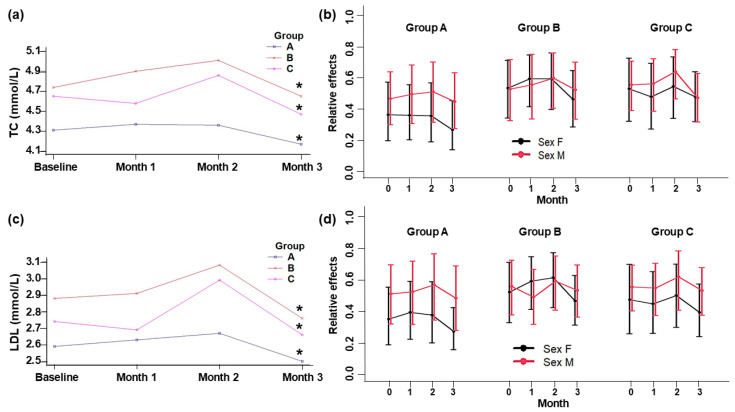
Levels of plasma total cholesterol (**a**) and LDL-cholesterol (**c**), and relative effects of plasma total cholesterol (**b**) and LDL-cholesterol (**d**) in adult subjects at baseline and 1, 2, and 3 months of intervention. Group A: Yogurt with SIO. Group B: Yogurt with HPO. Group C: Yogurt control (placebo). Data are means ± standard deviation. * Indicates a significant difference within groups (*p* ≤ 0.05).

**Figure 3:**
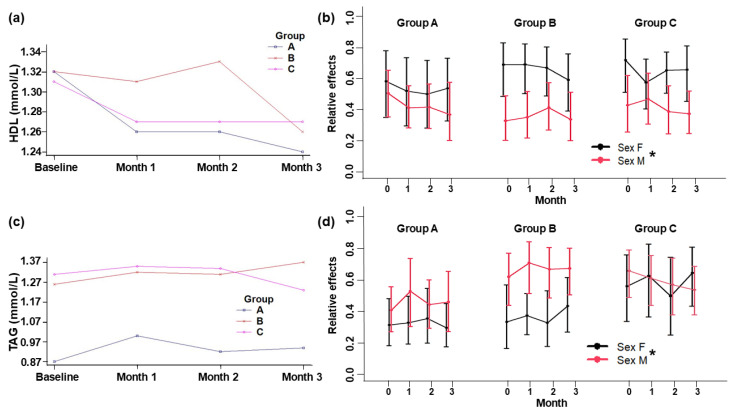
Plasma levels of HDL-cholesterol (**a**) and triacylglycerols (**c**). Relative effects of plasma HDL-cholesterol (**b**) and triacylglycerols (**d**) in adult subjects at baseline and 1, 2, and 3 months of intervention. Group A: Yogurt with SIO. Group B: Yogurt with HPO. Group C: Yogurt control (placebo). Data are means ± standard deviation. * Indicates a significant difference by sex (*p* ≤ 0.05).

The levels of plasma lipids from baseline to the end of the intervention remained within normal ranges (Figure 2 and Figure 3). No significant changes were detected between the control and treatment groups during the intervention. Nevertheless, there were relative within-group and between-sex intervention effects. TC and LDL-c plasma levels declined significantly at the end of the intervention period in all three groups (Figure 2), and, in women, the HDL-c was significantly higher (*p* = 0.003) and the TAG was significantly lower (*p* = 0.039) than in men in all groups (Figure 3). 

Although there were no significant differences between groups, within groups, the plasma TC (*p* < 0.001) and LDL-C (*p* = 0.001) levels decreased. Within Groups A and B, the values of these markers increased during the first and second months and decreased in the third one, whereas in Group C, the values increased in the second month and decreased in the first and third months. 

The levels of HDL-c did not differ between groups (*p* = 0.826). Within Group A, HDL-c levels showed a trend in reduction from baseline level; in Group B, there was a rise in the second month and a decrease in the third month; and in Group C, there was an HDL-c level reduction during the first month and then values remained stable (Figure 3). 

Regarding the plasma TAG levels (Figure 3), even though we did not find significant differences between groups (*p* = 0.118), TAG behavior showed changes within groups. The TAG levels rose by the first month of intervention; by the second month, they experienced a reduction; and by the end of the third month, these levels increased slightly in Groups A and B. In contrast, in Group C, the TAG levels remained stable the first two months and then decreased by the end of the third one. 

#### 3.2.2. Apolipoproteins A1, B and Their ApoB/ApoA1 Ratio 

**Figure 4:**
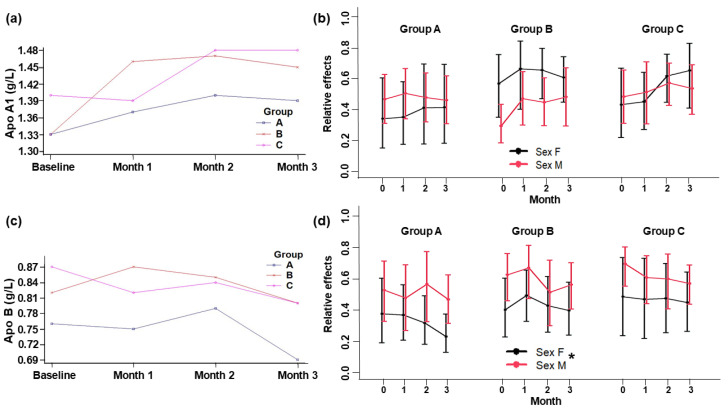
Plasma levels of Apolipoprotein A1 (**a**) and Apolipoprotein B (**c**). Relative effects of plasma Apolipoprotein A1 (**b**) and Apolipoprotein B (**d**) in adult subjects at baseline and 1, 2, and 3 months of intervention. Group A: Yogurt with SIO. Group B: Yogurt with HPO. Group C: Control yogurt (placebo). Data are means ± standard deviation. * Indicates a significant difference by sex (*p* ≤ 0.05).

**Figure 5:**
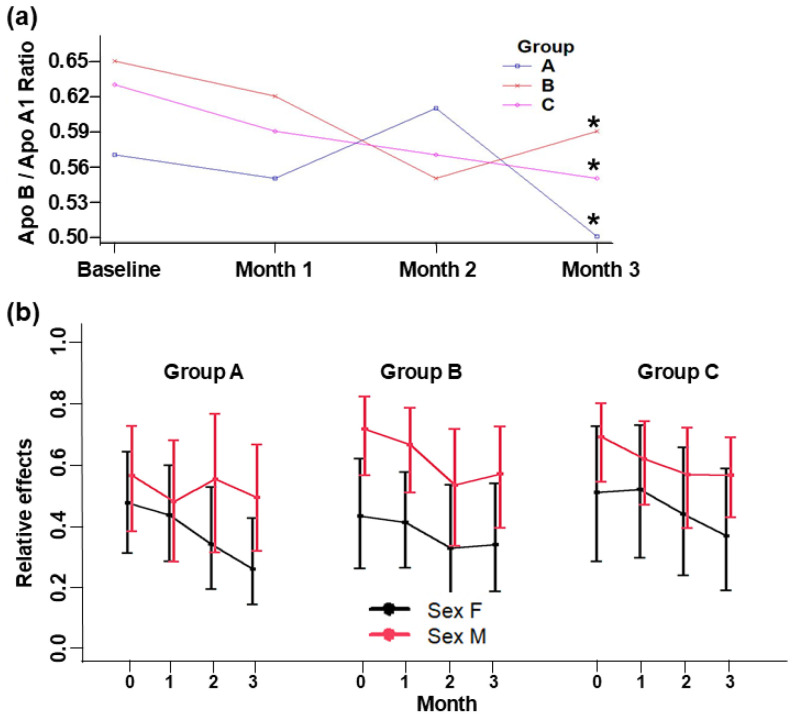
ApoB/ApoA1 ratios (**a**) and relative effects of ApoB/ApoA1 ratios (**b**) in adult subjects at baseline and 1, 2, and 3 months of intervention. Group A: Yogurt with SIO. Group B: Yogurt with HPO. Group C: Control yogurt (placebo). Data are means ± standard deviation. * Indicates a significant difference within groups (*p* ≤ 0.05).

Supplementation with yogurt did not show significant differences between the three groups for ApoA1 (*p* = 0.366), ApoB (*p* = 0.309), and the ApoB/ApoA1 ratio (*p* = 0.609). However, within groups, during the intervention, there was a significant difference in the ApoB/ApoA1 ratio (*p* < 0.001), and between sexes, there was a significant difference in Apo B (*p* = 0.021). Considering ApoA1 and ApoB behaviors, ApoA1 tended to increase, whereas ApoB decreased in all groups (Figure 4). ApoB was lower in Group A and decreased at the end of the intervention, whereas ApoA1 increased during the first two months and then remain stable until the end of intervention. In Group B, ApoB increased in the first month and then decreased, whereas ApoA1 increased in first month and then remained stable. In the control, Group C, ApoB decreased and ApoA1 decreased initially, but then increased to remain stable up to the end of the intervention. In all groups, ApoA1 was above of 1.10 g/L, which is the minimum normal level, and Apo B was below of 1.4 g/L, which is the maximum normal level. The ApoB/ApoA1 ratios significantly decreased within all groups (Figure 5). All subjects presented an ApoB/ApoA1 ratio below 0.65, which is normal for men (0.3–0.9) and women (0.35–1.0). This cardiovascular risk predictor was lower at the end of the intervention in Group A (~0.5) and higher in Groups C (~0.56) and B (~0.59). 

### 3.3. Dietary Intake 

All subjects who completed the study tolerated the yogurts and did not report side effects. The intake of MUFA, PUFA, and vitamin E from the normal diet differed significantly throughout the intervention period (Table 3); as expected, this intake was greater in Groups A and B than in Group C. Consequently, the total lipids and energy intake was significantly different between the three groups. Significant sex differences in protein, SFA, cholesterol, dietary fiber, Na, and K intake were also observed, being higher in males than in females. 

## 4. Discussion

### 4.1. Cardiovascular Disease Risk Markers: Lipid Profile 

Regarding Group A, the behavior of TC and LCL-c were similar to those obtained by Gonzales and Gonzales [6] with 10–15 mL of SIO supplementation, revealing a significant decrease in TC and LDL-c. However, the time frame effect differed between studies. While the present investigation identified a significant decrease between the second and third month within Group A, in the study using SIO alone, the reduction was evidenced during the first three months of intervention [6]. With respect to the yogurt supplemented with ALA, Hasaniani et al. [17] administer 200 g of yogurt (containing 30 g of ALA from flaxseed) to type 2 diabetes patients, showing a significant decrease in TC and a non-significant decrease in LDL-C after two months of intervention, resembling our findings; however, in the present study, our subjects were healthy. Similar results were obtained in a cohort study in which supplementation with a yogurt containing 2% of extruded flaxseed powders resulted in a significant reduction in TC and LDL-C after one month of intervention [18]. In the case of Group B, the behavior of TC and LDL-c resembled that of Group A, but the rise in TC and LDL-C was higher during first and second months. Considering that the fatty acid profile of the HPO-containing yogurt contains 3.21 ± 0.09 g of OA, our results agreed with Lucci et al. [19], who found that subjects supplemented with 25 mL (13.8 g of OA) of virgin HPO showed an increase in TC and LDL-c by the first month and a subsequent decrease by the end of the third month. However, they observed a more significant effect, probably due to direct supplementation with virgin HPO. In particular, tocotrienols from HPO have been shown to inhibit pathways involving activated B-cell kappa light chain, enhancing the nuclear factor [NF kβ], signal transducers and activators 3 [STAT3], and cyclooxygenase 2 [COX-2], which are critical pathways for triggering pathological inflammatory responses, in addition to exerting a hypocholesterolemic effect, which is possibly associated with an inhibitory activity of the enzyme hydroxymethylglutarylcoa reductase [HMGCoAR] hepatic reductase [11]. In the control group, the significant decrease in TC and LDL plasma levels could be attributed to the starter culture since *Lactobacillus acidophilus* and *Bifidobacterium lactis* are recognized for their probiotic activity. A recent metanalysis showed a significant reduction in LDL-c following probiotic yogurt consumption in subjects with mild-to-moderate hypercholesterolemia. Probiotics may regulate plasma lipids in the form of cholesterol ester along pathways related to its transport, either by promoting its excretion or by inhibiting its absorption via the inactivation of Niemann–Pick C1L1 (protein responsible for the movement of cholesterol into the enterocytes) [20].

With respect to HDL-c in the Group A values, similar results were obtained by Gonzales and Gonzales [6], where at the third month of intervention with SIO blood, HDL-c levels decreased; however, the behavior was different during the first and second months; in the present study, subjects supplemented with SIO-containing yogurt had decreased HDL-c levels in the first month of intervention. On the other hand, comparing the HDL-c in Group B with those reported by Lucci et al. [19], the results differed in the first month; whereas these authors detected a rise, we observed a reduction. Even though HDL-c levels decreased in the three groups, all levels remained within the normal range. This HDL-c reduction could also explain the TC reduction observed. Studies in which participants were supplemented with yogurts containing fish, canola, and *Dracocephalum ibericum* oils revealed a significant increase in HDL-c in subjects with hypertriglyceridemia and rheumatoid arthritis [21]. This effect could be attributed to the composition of the oils, since fish oil consists mainly of EPA and DHA. Because DHA relates better with HDL-c improvement than EPA, an increase in HDL-c can be explained by the enhanced lipoprotein lipase (LPL) activity [22]. Regarding Group C, our findings resemble those with probiotic yogurts since we did not find significant variation throughout the intervention within Group C. Nevertheless, the relative effects by sex were significant in all groups (Figure 3b). This may be due to physiological differences in lipid metabolism after puberty. These changes are observed mainly in women due to hormone shifts; for instance, liver estrogen signaling promotes the hepatic steps of reverse cholesterol transport [23]. However, Dawczynski et al. [24], who provided a yogurt with fish oil and rapeseed oil to hypertriglyceridemia subjects of both sexes, found that only women experienced an HDL-c reduction due to the inflammatory burden related to hypertriglyceridemia, countering the positive effects of PUFA [25]. HDL-c levels can also be influenced by physical activity which can become a more determining factor than diet itself. Aerobic exercise has been shown to trigger positive changes in HDL-c particle concentrations and functionality [26]; however, in the present study, the subjects maintained a moderate level (<30 min of activity per day that rose their heart rate by 60%). 

Observing plasma TAG levels, other clinical trials have shown a decrease in TAG following ALA intake. Soleimani et al. [27] administered 1000 mg of ALA per day via flaxseeds to 60 patients with diabetic nephropathy for 12 weeks, reporting a decrease in TAG levels by the end of the intervention. Comparable trials have also shown a decrease, with respect to baseline values, in TAG in patients with hypercholesterolemia following supplementation with 5 to 10 mL of SIO [28]. This suggests that supplementing healthy adults with SIO-containing yogurt, as in the present study, and providing 3.1 g of ALA could have a similar effect to that of 10 mL of SIO alone. Furthermore, Group A presented the lowest TAG levels throughout the intervention period compared with Groups B and C. The mechanism likely explaining the reduction in triglycerides due to ALA encompasses a decrease in the activity of the enzymes responsible for the synthesis of fatty acids, including the fatty acid synthetase, the Coa carboxylase, and the diacylglycerol acetyl transferase. In addition, this improves beta oxidation in the mitochondria and reduces the synthesis of TAGs and increases their catabolism [29]. Comparing TAG behavior within Group B with the literature, our results contrast with those of Lucci et al. [19]. During the first month, in which TAG experienced a reduction, we, on the contrary, observed an increase. At the end of the second month, our results were more comparable since TAG behavior tended to rise. Even so, our findings agree with Hay Yuen et al. [30], where volunteers supplemented with 300 mg of a mixture of palm tocotrienols showed an increase in TAG from the first month. Studies in mice have shown that OA can affect gene expression in the liver within the de novo synthesis of fatty acids and the inhibition of the Acetyl-Coa Carboxylase (ACC), thus being able to influence TAG concentrations in plasma [31]. The tocotrienols in HPO have shown a high lipid-lowering potential; however, depending on the isoform, their effect may vary [32]. HPO has four isoforms, of which approximately 73% correspond to the gamma (γ) and delta (δ) isoforms [5]. The γ and δ isoforms have the potential to suppress TAG biosynthesis in the liver by inhibiting Diacylglycerol O-acyltransferase 2 (DGAT2) in addition to increasing the expression of LDL-C receptors [33]. For this reason, the joint effect of these two HPO compounds may favor the decrease in TAG concentrations; this could explain the TAG behavior levels observed up to the second month. Furthermore, it has been observed that high-intensity aerobic exercise can reduce TAGs [34]; however, the subjects in our study had a mild to moderate level of physical activity, so it is likely that this variable had no effect on their lipid profile. In the case of Group C, our results agreed with the fact that conventional yogurt is associated with a decrease in plasma TAG due to probiotic activity exerted via the expression of the peroxisome proliferator-activated receptor-α (PPAR-α), carnitine palmitoyltransferase 2 (CPT 2), sterol regulatory element-binding protein-1 (SREBP-1), fatty acid synthase (FAS), and stearoyl-CoA desaturase-1 (SCD-1) [35]. Furthermore, the significant relative effects of sex within all groups (Figure 3d) could be attributed to the differences in TAG metabolism. TAG plasma levels were higher in men than in women, likely due to women having an improved clearance of meal-related TAGs and an enhanced fat storage in subcutaneous gluteal areas rather than in the abdomen, whereas fat storage in both areas (abdominal and subcutaneous) is equal in men [23]. 

### 4.2. Apolipoproteins A1, B and Their ApoB/ApoA1 Ratio 

Lipoprotein particles ApoB and ApoA1 are structural and functional components acting as cholesterol transporters. ApoB is present in LDL-C, whereas APOA1 is present in HDL-C; it has been suggested that an increase in Apo B concentrations and a decrease in Apo A1 concentrations are positively correlated with ischemic heart disease risk. So, the ApoB/ApoA1 ratio is a predictor of future cardiovascular events as Apo B and Apo A1 levels are unaffected by fasting status, as opposed to LDL-C [36]. Despite the absence of significant differences in our results, it is remarkable that regardless of treatment, ApoA1 had a total rise of 8.8%, 20.8%, and 6.6% in Groups A, B, and C, respectively (Appendix A and Appendix B). This behavior may be of interest since ApoA1 is related to the reverse transport of cholesterol, which reduces CVD risk. Recently, levels of ApoA1 above of 0.2 g/L have been associated with a 20% reduction in SARSCoV-2 infection risk [37].

ApoB decreased from basal to the end of the intervention by about 9.2%, 1.2%, and 6.8% in Groups A, B, and C, respectively (Appendix A and Appendix B). Even so, there were no significant differences among groups; Groups A and C had similar Apo B decrease rates, which can be beneficial since this apolipoprotein represents atherogenic particles [38]. Since ALA does not seem to affect the expression of ApoB [9], and this behavior was observed mainly in Groups A and C, it is likely that probiotics (content in the starter culture) could provoke an increase in circulating bile acid, which may have driven the observed reductions in plasma LDL-C and ApoB [20]. Furthermore, this reduction differed significantly between sexes; in Groups A and C, the relative effects tended to decrease in men. It is well established that men have a higher cardiovascular risk than women, so this reduction could be cardioprotective [23]. The behavior of ApoB/ApoA1 ratios suggest that SIO, HPO, and control yogurts may have a potential effect in early CVD prevention. According to Fernandez et al. [39] the bioactive lipids of yogurt, such as vaccenic acid, was reported to improve blood lipids via the activation of PPARα and PPARγ expression, and lauric acid is a potent cholesterol increaser, mainly increasing HDL-C and decreasing the total HDL-C cholesterol. This implies a lower risk of CVD since values closer to, or above, 1 have been reported in people with dyslipidemia, diabetes, and peritoneal dialysis, conditions that could develop into CVD co-morbidity [40,41,42]. More investigation must be conducted to elucidate the mechanism whereby these yogurts could affect Apo A1 and Apo B biomarkers. 

### 4.3. Dietary Intake 

In Group A, the ingestion of PUFA and vitamin E was significantly higher in months one to three compared to baseline values. In Group B, the ingestion of MUFA and vitamin E was slightly superior during the entire intervention period compared to baseline values. Among the three groups, PUFA, MUFA, and vitamin E intakes were significantly higher during intervention in Groups A and B than in Group C. It should be mentioned that in the third month of intervention, Group C increased their PUFA intake, mainly in males, probably due to the increase in the intake of foods prepared with sunflower oil (deep-fried potatoes and other starchy items), which was observed in the dietary records.

In this study, the volunteers maintained their usual food intake; the only restriction imposed was the consumption of any fermented milk other than the yogurts provided by this protocol. Regarding the consumption of MUFA, PUFA, and vitamin E, there were statistically significant differences between the groups, being higher during intervention in Groups A and B, due to the nutritional composition of the yogurt. This MUFA intake, mainly OA from plant origin provided in the yogurt with total fat replacement with HPO, may contribute to prevent long-term coronary heart disease [43]. As for omega 3 PUFA intake, there is substantial evidence highlighting their beneficial effect on CVD prevention [9].

A slight decrease in energy intake (yet not significant) was observed in Group A; it could be due to leptin’s appetite suppressant effect of ALA. Animal studies have shown an increase in leptin expression after supplementation with 20 mg of ALA [44]. On the other hand, it has been shown that ALA exerts effects on the endocannabinoid system, which has an important role in appetite regulation and in the metabolism of glucose and lipids. Pintus et al. [45] carried out a clinical trial in 42 adults with hypercholesterolemia, where they were given 90 g of ALA-enriched goat cheese for three weeks, evidencing a decrease in anandamide levels with a subsequent decrease in appetite, which could explain the slight decrease in energy intake for Group A. However, it is necessary to carry out more research to demonstrate the effect of daily ALA consumption of 3.1 g, as in our study. Furthermore, the increase in vitamin E in Groups A and B may lead to protection against CVD due to its antioxidant effect, also inhibiting platelet aggregation and thrombus formation. In the case of Group B, vitamin E, in the form of tocotrienols, exerts an hypolipidemic effect [11].

## 5. Conclusions

In the light of the current literature, the present study is the first to approach the functional potential of yogurts with total replacement of milk fat by SIO and HPO; therefore, its results prompt future investigation on the long-term effects of SIO and HPO yogurt supplementation. Following this study’s interventions, the participants’ lipid profile was improved in terms of TC and LDL-C, revealing a significant reduction after three months of daily yogurts consumption in all tested groups. This finding suggests that SIO, HPO, and plain yogurts may have a potential beneficial effect on plasma lipids, due to its share of fatty acids, such as ALA and OA in SIO- and HPO-containing yogurts, respectively, and its probiotics and nutritional quality. Moreover, HPO yogurt entails more vitamin E than SIO yogurt, which can explain its slightly better effect on TC and LDL; HPO tocotrienols have a well-known hypolipidemic effect. ApoB/ApoA1 ratios showed a tendency to decrease, which may be assumed as a beneficial effect on CVD risk.

Furthermore, the SIO- and HPO-containing yogurts of the present study resulted in a strategy to significantly improve the intake of MUFA (OA), PUFA (ALA), and vitamin E. Finally, we demonstrated that the daily consumption of the three yogurts (SIO, HPO, and the full milk fat control) without added sugars did not alter the body composition of healthy individuals. These results suggest that these yogurts do not alter the participant’s lipid profile and apolipoproteins A1 and B levels, since all parameters in these healthy subjects remained within normal ranges. This prompts future investigation in subjects with pathology conditions such dyslipidemia, hypertension, and inflammatory diseases to determine the yogurt behavior in this population with altered metabolisms.

## Figures and Tables

**Table 1:** Description of nutritional composition of a 200 g serving of the yogurts prepared in this trial.

Composition (200 g)	Yogurt		
	Control *	SIO *	HPO **
Energy (kJ)	542.6 ± 27.06	554.98 ± 6.86	576.16 ± 31.6
Protein (g)	7.48 ± 0.30	9.18 ± 0.64	7.66 ± 0.64
Carbohydrates (g)	10.18 ± 1.19	8.66 ± 1.00	11.24 ± 1.88
Total lipids (g)	6.58 ± 0.14	6.82 ± 0.38	6.90 ± 0.34
SFA (g)	4.72 ± 0.14	0.68 ± 0.02	2.48 ± 0.07
MUFA (g)	1.78 ± 0.05	0.58 ± 0.01	3.22 ± 0.06
OA (g)	1.66 ± 0.07	0.57 ± 0.01	3.21 ± 0.09
PUFA (g)	0.08 ± 0.00	5.57 ± 0.02	0.70 ± 0.01
ALA (g)	0.00	3.10 ± 0.05	0.00
Vitamin E (mg)	0.66 ± 0.01	0.42 ± 0.16	2.44 ± 0.02

SFA: Saturated fatty acids. MUFA: Monounsaturated fatty acids. PUFA: Polyunsaturated fatty acids. OA: Oleic Acid. ALA: Alpha linolenic acid. SIO: Sacha Inchi Oil. HPO: Interspecific Hybrid Palm Oil * Villamil et al. [5]. ** Unpublished data.

**Table 2:** Baseline and intervention characteristics of the study population.

Group		A				B				C				
Parameter		Males	Females	Total ^3^	*p* Value ^1^	Males	Females	Total ^3^	*p* Value ^1^	Males	Females	Total ^3^	*p* Value ^1^	*p* Value ^2^
Sex (n)		8	7	15		9	9	18		8	6	14		
Age (years)		25.9 ± 9.6	22.4 ± 6.9	24.3 ± 8.7		31.3 ± 8.1	24.8 ± 8.6	27.9 ± 8.9		28.3 ± 7.7	21.7 ± 3.9	25.4 ± 7.1		0.20
Weight (kg)	Baseline	75.0 ± 8.7	62.8 ± 6.6	69.0 ± 9.8	0.46	72.6 ± 4.2	58.9 ± 8.1	65.7 ± 9.4	1.00	75.3 ± 12.1	58.0 ± 9.6	66.7 ± 14.0	0.78	0.64
	3 Mos.	74.9 ± 9.0	62.5 ± 6.5	68.7 ± 10.1		72.6 ± 4.0	58.8 ± 8.5	65.7 ± 9.4		74.8 ± 11.9	58.4 ± 10.1	66.6 ± 13.8		
WC (cm)	Baseline	84.8 ± 6.3	75.2 ± 5.9	80.3 ± 7.7	0.43	82.4 ± 3.2	74.2 ± 5.6	78.3 ± 6.2	0.36	84.9 ± 9.5	71.1 ± 9.7	79.0 ± 11.8	0.39	0.62
	3 Mos.	84.5 ± 6.5	74.9 ± 5.9	80.0 ± 7.8		83.0 ± 3.7	73.4 ± 5.1	78.2 ± 6.5		84.7 ± 9.0	70.6 ± 9.7	78.6 ± 11.7		
BMI (kg/m^2^)	Baseline	25.0 ± 2.3	23.4 ± 2.3	24.3 ± 2.4	0.48	24.0 ± 1.6	23.6 ± 3.4	23.8 ± 2.6	0.48	25.2 ± 3.5	22.4 ± 3.3	24.0 ± 3.7	0.87	0.88
	3 Mos.	25.0 ± 2.4	23.2 ± 2.3	24.2 ± 2.5		24.0 ± 1.3	23.5 ± 3.3	23.7 ± 2.6		25.1 ± 3.4	22.5 ± 3.5	24.0 ± 3.7		
FMI (kg/m^2^)	Baseline	6.6 ± 1.4	7.9 ± 1.3	7.2 ± 1.5	0.71	5.6 ± 1.0	7.6 ± 2.4	6.6 ± 2.1	0.25	6.3 ± 2.2	6.6 ± 2.3	6.5 ± 2.2	0.69	0.30
	3 Mos.	6.5 ± 1.5	7.9 ± 1.3	7.2 ± 1.5		5.4 ± 1.0	7.5 ± 2.3	6.5 ± 2.1		6.3 ± 2.1	6.6 ± 2.3	6.4 ± 2.2		
FFMI (kg/m^2^)	Baseline	18.6 ± 1.2	15.5 ± 1.1	17.1 ± 1.9	0.35	18.5 ± 1.2	16.0 ± 1.2	17.2 ± 1.7	0.40	18.9 ± 1.5	15.7 ± 1.2	17.5 ± 2.1	0.83	0.64
	3 Mos.	18.5 ± 1.2	15.4 ± 1.1	17.0 ± 1.9		18.6 ± 1.1	16.0 ± 1.4	17.3 ± 1.8		18.8 ± 1.6	16.0 ± 1.3	17.6 ± 2.0		
SysP (mmHg)	Baseline	128.3 ± 11.6	123.6 ± 6.5	126.1 ± 9.9	0.48	124.6 ± 7.3	118.7 ± 12.3	121.5 ± 10.5	0.66	125.6 ± 9.1	119.3 ± 5.2	122.9 ± 8.3	0.01	0.11
	3 Mos.	125.9 ± 8.8	122.3 ± 11.2	124.2 ± 10.2		129.1 ± 9.9	111.1 ± 10.2	120.1 ± 13.5		117.8 ± 10.3	112.3 ± 10.1	115.4 ± 10.6		
DiaP (mmHg)	Baseline	72.6 ± 5.9	67.3 ± 5.5	70.1 ± 6.3	0.85	73.9 ± 9.4	71.1 ± 6.5	72.4 ± 8.2	0.20	74.5 ± 10.0	63.8 ± 6.6	69.9 ± 10.2	0.53	0.58
	3 Mos.	72.5 ± 4.0	66.9 ± 6.7	69.9 ± 6.1		66.2 ± 9.2	66.4 ± 6.2	69.4 ± 6.9		70.9 ± 7.3	65.2 ± 7.2	68.4 ± 7.8		

Values shown are means ± standard deviation. SysP = systolic pressure; DiaP = diastolic pressure. ^1^ = *p* value within groups. ^2^ = *p* value between groups. ^3^ = total average between males and females, Group A: Yogurt with 3.5% of Sacha Inchi oil (SIO). Group B: Yogurt with 3.5% of hybrid palm oil (HPO). Group C: Control yogurt (placebo). Mos = months; WC = waist circumference; BMI = body mass index; FMI = fat mass index; FFMI = fat-free mass index.

**Table 3:** Consumption of nutrient according to 24 h recall and dietary records at baseline (0) and 1, 2, and 3 months of intervention.

	Months	0		1		2		3	
Parameter	Group	Mean Sd	*p* Value	Mean Sd	*p* Value	Mean Sd	*p* Value	Mean Sd	*p* Value
Energy (kcal)	A	1771.0 ± 593.9	0.632	1964.1 ± 660.5	0.768	1829.7 ± 708.1	0.018	1820.6 ± 588.6	0.290
	B	1850.4 ± 652.9		2073.1 ± 668.5		2118.1 ± 737.7		2027.1 ± 618.0	
	C	2056.3 ± 619.7		2055.3 ± 736.9		1837.7 ± 775.7		2042.4 ± 791.1	
Protein (g)	A	74.2 ± 30.5	0.836	84.9 ± 31.1	0.007	79.7 ± 30.4	0.003	81.8 ± 28.0	0.293
	B	79.2 ± 29.4		96.3 ± 35.9		94.8 ± 35.1		90.6 ± 26.5	
	C	80.6 ± 28.5		96.9 ± 34.1		83.9 ± 32.7		101.5 ± 46.0	
Total lipids (g)	A	67.9 ± 24.6	0.914	76.8 ± 34.1	0.410	72.5 ± 36.2	0.004	73.9 ± 35.3	0.273
	B	73.7 ± 36.6		81.3 ± 38.4		88.6 ± 39.5		84.1 ± 30.5	
	C	77.4 ± 39.1		84.3 ± 38.7		73.5 ± 35.5		90.3 ± 40.1	
SFA (g)	A	18.4 ± 7.5	0.904	19.7 ± 9.5	0.001	19.3 ± 11.1	0.002	17.6 ± 8.4	<0.001
	B	17.5 ± 6.9 ^a^		23.1 ± 11.3 ^ab^		25.1 ± 12.8 ^ab^		27.9 ± 15.9 ^b^	
	C	19.7 ± 10.3		25.9 ± 11.3		22.4 ± 11.9		29.1 ± 10.5	
MUFA (g)	A	18.3 ± 9.8	0.881	21.4 ± 13.7	0.004	19.8 ± 12.0	0.092	18.7 ± 9.7	0.002
	B	22.1 ± 15.3 ^a^		27.2 ± 18.6 ^ab^		30.6 ± 15.8 ^ab^		28.0 ± 11.8 ^b^	
	C	21.2 ± 15.8		25.8 ± 17.4		22.5 ± 13.9		28.3 ± 15.5	
PUFA (g)	A	9.7 ± 7.6 ^a^	0.450	14.9 ± 8.5 ^b^	0.037	16.6 ± 10.1 ^b^	0.003	14.4 ± 6.4 ^b^	0.413
	B	14.7 ± 14.8		13.8 ± 10.8		16.6 ± 11.1		13.3 ± 7.1	
	C	10.6 ± 13.0		13.7 ± 10.9		11.3 ± 11.0		16.3 ± 13.6	
Cholesterol (mg)	A	517.3 ± 414.9	0.379	462.5 ± 281.3	0.582	468.4 ± 339.7	0.790	349.9 ± 287.2	0.011
	B	393.1 ± 268.3		467.1 ± 364.5		451.5 ± 319.6		469.3 ± 213.2	
	C	371.6 ± 207.1		430.7 ± 311.8		427.8 ± 254.1		593.0 ± 246.6	
Carbohydrates (g)	A	209.4 ± 74.3	0.274	222.3 ± 78.5	0.573	201.5 ± 79.2	0.104	198.6 ± 64.9	0.471
	B	207.7 ± 93.6		227.9 ± 87.8		223.1 ± 94.5		218.1 ± 80.7	
	C	252.1 ± 69.8		217.4 ± 85.5		198.3 ± 98.9		192.5 ± 73.1	
Dietary fiber (g)	A	9.9 ± 5.4	0.059	14.0 ± 9.8	0.267	11.2 ± 6.5	0.026	11.9 ± 5.3	0.035
	B	16.3 ± 11.4		16.3 ± 9.7		15.2 ± 11.1		16.9 ± 8.1	
	C	18.4 ± 10.2		15.8 ± 10.1		13.2 ± 6.1		14.1 ± 7.2	
Vitamin E (mg)	A	0.05 ± 0.14 ^a^	0.912	0.67± 0.64 ^b^	<0.001	0.53 ± 0.52 ^b^	<0.001	0.45 ± 0.36 ^b^	<0.001
	B	0.18 ± 0.04 ^a^		2.44 ± 0.00 ^b^		2.38 ± 0.37 ^b^		2.38 ± 0.34 ^b^	
	C	0.26 ± 0.15		0.32 ± 0.11		0.34 ± 0.28		0.38 ± 0.19	
Sodium (mg)	A	1746.7 ± 938.5	0.093	2255.0 ± 1347.0	0.068	2372.1 ± 1572.0	0.004	2003.6 ± 933.0	0.015
	B	1955.0 ± 990.8		2581.2 ± 1681.6		2928.6 ± 1655.6		2833.5 ± 1411.9	
	C	2481.3 ± 879.5		2187.1 ± 1021.4		2036.3 ± 1027.1		2692.2 ± 1050.7	

Values are means ± standard deviation (sd). Different lowercase letters in a column indicate a significant difference within groups (*p* ≤ 0.05). Group A: Yogurt with 3.5% of Sacha Inchi oil (SIO). Group B: Yogurt with 3.5% of hybrid palm oil (HPO). Group C: Control yogurt (placebo).

## Data Availability

The original contributions presented in the study are included in the article, further inquiries can be directed to the corresponding author.

## Appendix A

**Table A1:** Yogurt’s total LAB count and safety parameters for microbiological quality.

Yogurt	Storage Time (days)	Total LAB Count	Total Coliforms (g)	Fecal Coliforms (g)	Coagulase Staphylococcus (+) ufc/g	Mold and Yeast Count ufc/g
A	1	2.3 × 10^7^	<10	<1	<10	100
	21	4.3 × 10^7^	<10	<1	<10	300
B	1	2.8 × 10^7^	<10	<1	<10	100
	21	2.0 ×10^7^	<10	<1	<10	100
C	1	6.8 × 10^7^	<10	<1	<10	50
	21	3.0 × 10^7^	<10	<1	<10	80
Normal range			20–93	<3	-	200–500

Group A: Yogurt with SIO. Group B: Yogurt with HPO. Group C: Control yogurt (placebo). LAB: lactic acid bacteria.

## Appendix B

**Table A2:** Overall variation from baseline by month for lipid profile, Apo A1, Apo B, and ApoB/ApoA1 ratio.

Variables	Months	Group A		Group B		Group C	
		Variation	Conf. Int. (95%)	Variation	Conf. Int. (95%)	Variation	Conf. Int. (95%)
TC (mmol/L)	1	1.6	−1.28 to 4.60	3.6	0.80 to 6.39	−1.4	−5.46 to 2.74
	2	1.2	−1.76 to 4.12	5.9	3.08 to 8.66	4.8	0.71 to 8.92
	3	−2.8	−5.71 to 0.18	−1.3	−4.06 to 1.51	−3.3	−7.42 to 0.78
LDL-C (mmol/L)	1	2.0	−3.34 to 7.31	2.1	−2.34 to 6.49	−2.5	−9.54 to 4.41
	2	4.2	−1.12 to 9.52	8.0	3.61 to 12.44	8.8	1.85 to 15.81
	3	−1.6	−6.94 to 3.71	−2.5	−6.96 to 1.87	−2.0	−9.00 to 4.94
HDL-C (mmol/L)	1	−4.3	−6.95 to −1.69	0.0	−3.62 to 3.66	−2.3	−6.12 to 1.58
	2	−4.1	−6.69 to −1.43	1.6	−2.01 to 5.27	−2.9	−6.79 to 0.91
	3	−5.8	−8.45 to −3.19	−3.6	−7.24 to 0.04	−3.3	−7.18 to 0.52
TAG (mmol/L)	1	11.1	−0.63 to 22.72	14.9	3.11 to 26.73	3.1	−7.46 to 13.68
	2	4.9	−6.82 to 16.54	9.4	−2.43 to 21.188	−2.2	−12.81 to 8.33
	3	7.7	−3.94 to 19.41	18.9	7.12 to 30.7435	−1.6	−12.21 to 8.93
Apo A1 (g/L)	1	6.9	−3.4241 to 17.13	16.3	−2.45 to 35.08	0.5	−4.47 to 5.53
	2	7.9	−2.35 to 18.20	22.6	3.84 to 41.38	6.7	1.73 to 11.75
	3	8.4	−1.88 to 18.67	20.8	2.02 to 39.57	6.6	1.64 to 11.66
Apo B (g/L)	1	2.5	−13.01 to 18.04	11.1	−8.97 to 31.24	−5.1	−12.50 to 2.30
	2	11.4	−4.13 to 26.92	14.3	−5.81 to 34.41	−2.7	−10.14 to 4.66
	3	−1.9	−17.43 to 13.62	6.1	−13.98 to 26.23	−5.6	−12.969 to 1.84
ApoB/ApoA1 ratio	1	−4.2	−17.36 to 9.04	−3.5	−7.48 to 0.51	−5.2	−10.76 to 0.45
	2	5.3	−7.86 to 18.54	−10.8	−14.81 to −6.82	−8.6	−14.27 to −3.05
	3	−9.7	−22.94 to 3.45	−11.8	−15.79 to −7.79	−11.7	−17.31 to −6.09

TAG, triacylglycerol; TC, total cholesterol, HDL-C, high-density lipoprotein cholesterol, and LDL-C low-density cholesterol; Apo A1, Apo B, apolipoproteins A1 and B. Group A: Yogurt with SIO. Group B: Yogurt with HPO. Group C: Control yogurt (placebo).
